# Evaluating the Diagnostic Potential of Combined Salivary and Skin Biomarkers in Parkinson’s Disease

**DOI:** 10.3390/ijms25094823

**Published:** 2024-04-28

**Authors:** Matteo Costanzo, Eleonora Galosi, Maria Ilenia De Bartolo, Gaetano Gallo, Giorgio Leodori, Daniele Belvisi, Antonella Conte, Giovanni Fabbrini, Andrea Truini, Alfredo Berardelli, Giorgio Vivacqua

**Affiliations:** 1 Department of Human Neuroscience, Sapienza University of Rome, Viale dell’Università 30, 00185 Rome, Italy; matteo.costanzo@uniroma1.it (M.C.); eleonora.galosi@uniroma1.it (E.G.); giorgio.leodori@uniroma1.it (G.L.); antonella.conte@uniroma1.it (A.C.); giovanni.fabbrini@uniroma1.it (G.F.); andrea.truini@uniroma1.it (A.T.); alfredo.berardelli@uniroma1.it (A.B.); 2 Department of Neuroscience, Istituto Superiore di Sanità, Viale Regina Elena 299, 00161 Rome, Italy; 3 IRCCS Neuromed, Via Atinense 18, 86077 Isernia, Italy; mariailenia.debartolo@uniroma1.it; 4 Unità Operativa Complessa Neurologia, Ospedali Riuniti Padova Sud, Via Albere 30, 35043 Padova, Italy; gaetano.gallo@uniroma1.it; 5 Department of Experimental Morphology and Microscopy-Integrated Research Center (PRAAB), Campus Biomedico University of Rome, 00128 Rome, Italy; g.vivacqua@unicampus.it

**Keywords:** Parkinson’s disease, biomarkers, synucleinopathies, alpha-synuclein

## Abstract

Oligomeric alpha-synuclein (α-syn) in saliva and phosphorylated α-syn deposits in the skin have emerged as promising diagnostic biomarkers for Parkinson’s disease (PD). This study aimed to assess and compare the diagnostic value of these biomarkers in discriminating between 38 PD patients and 24 healthy subjects (HSs) using easily accessible biological samples. Additionally, the study sought to determine the diagnostic potential of combining these biomarkers and to explore their correlations with clinical features. Salivary oligomeric α-syn levels were quantified using competitive ELISA, while skin biopsies were analyzed through immunofluorescence to detect phosphorylated α-syn at Ser129 (p-S129). Both biomarkers individually were accurate in discriminating PD patients from HSs, with a modest agreement between them. The combined positivity of salivary α-syn oligomers and skin p-S129 aggregates differentiated PD patients from HSs with an excellent discriminative ability with an AUC of 0.9095. The modest agreement observed between salivary and skin biomarkers individually suggests that they may reflect different aspects of PD pathology, thus providing complementary information when combined. This study’s results highlight the potential of utilizing a multimodal biomarker approach to enhance diagnostic accuracy in PD.

## 1. Introduction

The diagnosis of Parkinson’s disease (PD) is currently based on clinical criteria, with inadequate sensitivity, especially at early disease stages [1,2]. One of the main pathological features of PD is the abnormal deposition of misfolded α-synuclein (α-syn), leading to neuronal dysfunction and degeneration [3,4,5,6,7]. In PD, α-syn monomers aggregate into neurotoxic α-syn oligomers, which are then converted into insoluble fibrils and deposited into Lewy bodies and neurites, the histopathological disease hallmark [7,8]. The misfolding of α-syn can be facilitated by several factors, including post-translational modifications of the protein structure, like the phosphorylation at specific aminoacidic residues, including serine at position 129 (p-S129) or 87 (p-S87) [7]. In the last decade, an increasing effort has been dedicated to disease biomarker identification, with α-syn being the most widely investigated [9,10,11,12,13,14,15]. The earliest studies, which focused on the analysis of cerebrospinal fluid (CSF), demonstrated that concentrations of oligomeric α-syn in the CSF are higher in PD patients compared to control subjects, as determined by immunoassays such as ELISA [13,14,16,17,18,19,20]. More recently, these findings have been confirmed using more accessible biological fluids, including plasma [21,22] and saliva [9,11,12,23,24], suggesting that the increase in oligomeric α-syn in these samples may serve as a potential molecular biomarker of PD. At the same time, the detection of intra-neural pathologically phosphorylated isoforms of α-syn deposits in the skin has been shown to reliably distinguish patients with PD from healthy controls as well as from those with atypical parkinsonism [25,26,27,28]. Recent studies employing seeding assay techniques, including real-time quaking-induced conversion (RT-QuIC) and protein misfolding cyclic amplification (PMCA), have confirmed that various samples, including CSF [29,30,31], blood [32], skin [33,34] and saliva [10,35], exhibit a greater α-syn seeding capacity in PD patients compared to healthy subjects (HSs), thus providing an indirect confirmation of the utility of α-syn detection in biological fluids and tissues for diagnostic purposes. Although the diagnostic utility of α-syn detection in the abovementioned samples has been widely tested, direct comparisons between the effectiveness of multiple detection techniques have only been conducted in isolated cases [36,37]. To our knowledge, only one study has assessed the in vivo distribution of α-syn across multiple biofluids such as saliva, blood or CSF and peripheral tissues, including skin and submandibular biopsies, in patients with PD [37]. However, in this study, only total α-syn has been detected in biofluids, without a specific determination of oligomeric and aggregated species [37]. In this cross-sectional study, we aimed to compare the diagnostic value of salivary α-syn oligomers and p-S129 α-syn skin deposits in discriminating between 38 PD patients and 24 HSs who underwent detailed clinical phenotyping, saliva collection and skin biopsy sampling. Our findings will help to further confirm the diagnostic yield of these two promising techniques for α-syn detection, to determine the agreement degree between the methods and to understand whether their combination may be profitable. Furthermore, our study will improve our understanding of PD pathophysiological mechanisms by clarifying whether the α-syn oligomer concentration in biological fluids such as saliva reflects the extent of α-syn intra-neural aggregation in peripheral tissues like skin.

## 2. Results

### 2.1. Demographic and Clinical Characteristics of PD Patients and Healthy Subjects

PD patients were comparable to HSs in terms of gender (χ^2^ = 2.796; df = 1; *p* = 0.0946), as demonstrated by a chi-square analysis. Conversely, age was significantly different between the two groups, being higher in the PD group (t = 3.926; df = 60; *p* = 0.0002). Clinical and demographic features of PD patients and HSs are reported in Table 1.

### 2.2. Salivary Oligomeric α-Synuclein

**Figure 1:**
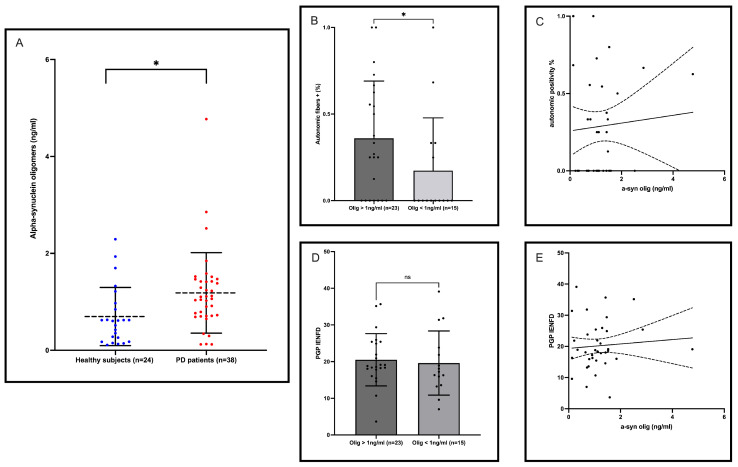
Salivary oligomeric alpha-synuclein in PD patients. (**A**) Comparison of salivary oligomeric α-syn levels between PD patients and healthy subjects. (**B**) Comparison, by using the Mann–Whitney U test, of p-S129 α-synuclein deposition in autonomic skin fibers between PD patients based on salivary α-synuclein oligomer concentrations. (**C**) Simple linear regression analysis between p-S129 α-synuclein deposition in autonomic skin fibers and salivary α-synuclein oligomer concentrations (*p* = 0.709; R squared = 0.004). (**D**) Comparison of intraepidermal nerve fiber density, assessed by using PGP9.5 immunofluorescence, between PD patients based on salivary α-synuclein oligomer concentrations. (**E**) Simple linear regression analysis between intraepidermal nerve fiber density and salivary α-synuclein oligomer concentrations (*p* = 0.65; R squared = 0.005). Mean values (dotted lines) and standard deviations (error bars) are shown in Figure 1**A**,**B**,**D**. Line of best fit and confidence interval (dotted lines) are shown in Figure 1**C**,**E**. The asterisk (*) indicates statistical significance.

**Figure 2:**
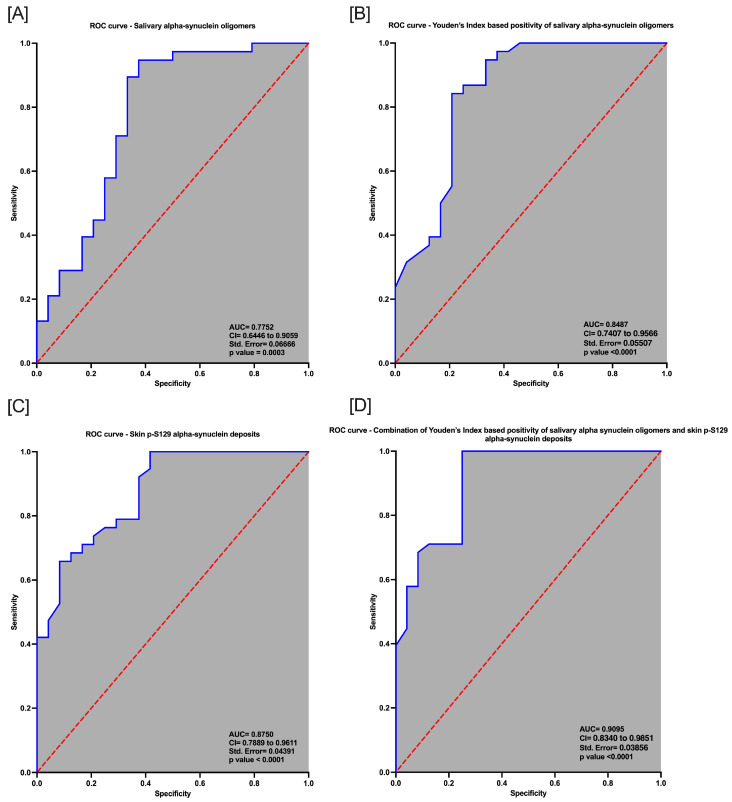
Receiver operating characteristic (ROC) curve analysis. Receiver operating characteristic (ROC) curves for the prediction of Parkinson’s disease based on salivary oligomeric alpha-synuclein values (**A**), Youden’s index-based positivity of salivary alpha-synuclein oligomers (**B**), presence/absence of skin p-S129 alpha-synuclein deposits (**C**) or the combination of Youden’s index-based positivity of salivary alpha-synuclein oligomers and skin p-S129 alpha-synuclein deposits (**D**).

The Mann–Whitney U test revealed that oligomeric α-syn levels were significantly higher in the saliva of PD patients than in HSs (U = 256; *p* = 0.0133) (Figure 1A). The mean values of salivary oligomeric α-syn in PD patients and HSs are outlined in Table 2. The Spearman correlation analysis demonstrated that salivary α-syn oligomers did not correlate with demographic measures, including age (*p* = 0.718; r = 0.060) or disease duration (*p* = 0.270; r = 0.184), or clinical measures in PD patients. Similarly, no correlation was found between salivary α-syn oligomers and age in HSs (*p* = 0.659; r = 0.095) (See Supplementary Materials). An age and gender-adjusted logistic regression model using salivary oligomeric α-syn values as an independent variable significantly predicted the diseased/healthy subject state status, as a binary outcome variable, in participants (*p* = 0.0003; 95% CI = 0.6446 to 0.9059; std error = 0.06666), with a moderate diagnostic ability indicated by an area under the ROC curve (AUC) of 0.7752 (negative predictive power: 78.95%; positive predictive power: 79.07%) (Figure 2A). The diagnostic test characteristics varied across different cut-off points, reflecting a trade-off between sensitivity and specificity. Notably, the highest Youden’s index was observed at a cut-off value of >0.6387, where the test exhibited a sensitivity of 80%, specificity of 80% and a positive likelihood ratio of 2.4. Therefore, we designed an age and gender-adjusted logistic regression model using the Youden’s index-based positivity of salivary α-syn oligomers as an independent variable to predict the diseased/healthy subject state status, as a binary outcome variable, in participants. We found that the model discriminated between patients and HSs with an AUC of 0.8487 (*p* < 0.0001; 95% CI = 0.7407 to 0.9566; std error = 0.05507; negative predictive power: 78.26%; positive predictive power: 84.62%) (Figure 2B).

### 2.3. Skin Biopsy Detection of p-S129 α-syn Deposits and Intraepidermal Nerve Fiber Density

**Figure 3:**
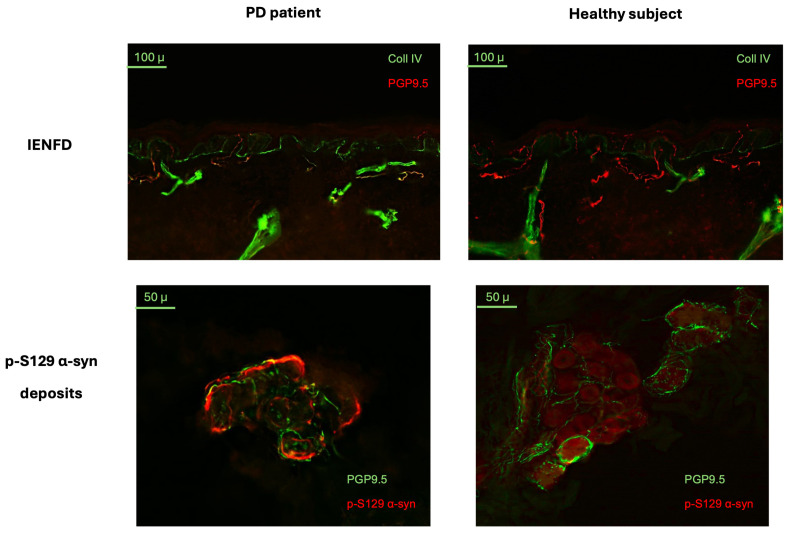
Skin biopsy findings in patients with Parkinson’s disease and healthy subjects. Exemplificative images from PD patients and healthy subjects. In the images representing intraepidermal nerve fiber density (IENFD), nerve fibers are marked in red by protein gene product 9.5 (PGP9.5), whereas the basal membrane is stained in green by collagen IV. Patients presented lower IENFD compared to healthy controls. In the pictures outlining p-S129 α-syn deposits, nerve fibers are stained in green by PGP9.5, and p-S129 α-syn is marked in red. PD patients more frequently presented p-S129 α-syn aggregates in dermal annexes like sweat glands.

**Figure 4:**
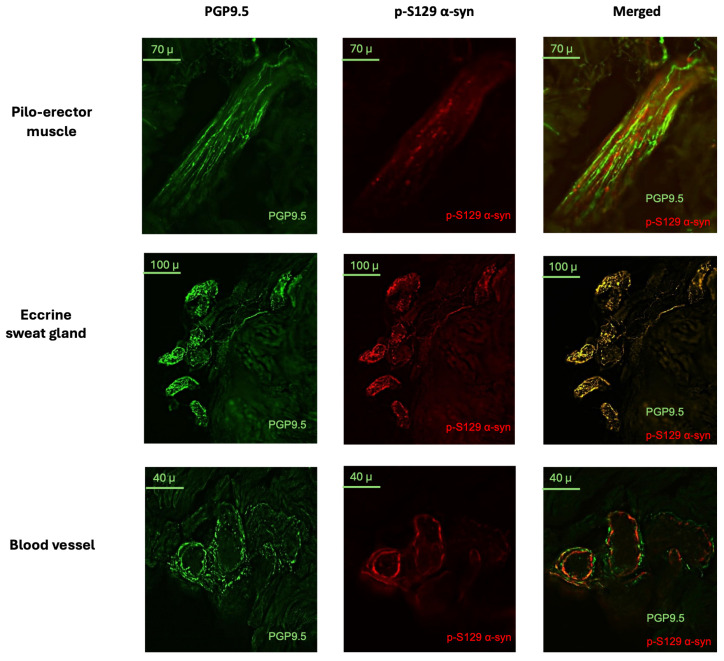
p-S129 alpha-synuclein deposits in dermal annexes in PD patients. Exemplificative images from PD patients with positive detection of p-S129 α-syn aggregates in dermal annexes, like piloerector muscles, eccrine sweat glands and vascular structures. Dermal nerve fibers are marked in green by the pan-axonal marker protein gene product 9.5, PGP9.5; p-S129 α-syn deposits are marked in red. Only p-S129 α-syn deposits that colocalized with PGP9.5 were considered positive, as shown in the merged images.

The main skin biopsy outcome variables are outlined in Table 2 for both PD patients and HSs. Intraepidermal nerve fiber density was significantly lower in patients with PD than in HSs (U = 210; *p* = 0.0008) (Figure 3) and was negatively correlated with age in PD patients (*p* = 0.0170; r = −0.2410). P-S129 α-syn deposits were detected in autonomic structures, i.e., sweat glands, piloerector muscles, blood vessels and/or in dermal nerve bundles, in 26/38 (68%) of patients and 2/24 (8%) of HSs (Figure 4). Patients with p-S129 α-syn deposits in the skin showed lower densities of intraepidermal nerve fibers (U = 80; *p* = 0.0050) and had higher UPDRS III (U = 86; *p* = 0.0065) and NMSS total scores (U = 75.5; *p* = 0.0036) compared to those without deposits (See Supplementary Materials). An age- and gender-adjusted logistic regression model with the diseases/healthy subject state as a binary outcome variable and the presence/absence of p-S129 α-syn deposits as an independent variable showed that the presence of p-S129 α-syn deposits was significantly associated with the diseased state (AUC = 0.8750; 95% CI = 0.7889–0.9611; *p* < 0.0001; std error = 0.04391; negative predictive power: 68.18%; positive predictive power: 77.5%) (Figure 2C).

### 2.4. Comparison between Skin-and Saliva-Based Biomarkers and Diagnostic Accuracy of Combined Detection of Salivary Alpha-Synuclein Oligomers and p-S129 α-syn Aggregates in Skin Biopsy

Overall, 26/38 (68%) PD patients and 2/24 (8%) HSs had detectable deposits of p-S129 α-syn in the skin, whereas 32/38 (84%) PD patients and 7/24 (29%) HSs had higher oligomeric α-syn levels with respect to the most accurate cut-off value, as identified by the Youden’s index. A total of 21/38 (55%) PD patients had both p-S129 α-syn deposits and high oligomeric α-syn levels in the saliva (double positivity), 11/38 (29%) had only high oligomeric α-syn levels and 5/38 (13%) PD patients only had p-S129 α-syn deposits in the skin. A total of 15/24 (63%) HSs had neither p-S129 α-syn deposits nor high oligomeric α-syn levels in the saliva (double negativity), 2/24 (8%) only had skin biopsy positive for p-S129 α-syn deposits and 7/24 (29%) had isolated elevation of salivary α-syn oligomers. The agreement between saliva and skin α-syn detection, as evaluated by Cohen’s kappa index, resulted to be modest (% of agreement: 59.02%, Cohen’s k: 0.199). No correlations were found by using the Spearman rank correlation coefficient between salivary oligomeric α-syn levels and p-S129 α-syn skin positivity (*p* = 0.088; r = 1.000) in PD patients. Notably, a distinct observation emerged for PD patients with an α-syn oligomer concentration exceeding 1 ng/mL. Interestingly, this subgroup presented an increased deposition of p-S129 α-syn in the autonomic fibers of the skin, as demonstrated by using the Mann–Whitney U test (U = 109; *p* = 0.01) (Figure 1B,C). Conversely, oligomeric α-syn in saliva did not demonstrate any relation with the intraepidermal nerve fiber density, as measured by PGP9.5 immunofluorescence (Figure 1D,E). Thereafter, we evaluated, by designing an age- and gender-adjusted logistic regression analysis, the diagnostic yield of the combined positivity of the two tests, i.e., the presence of skin p-S129 α-syn deposits combined with the detection of higher salivary oligomeric α-syn with respect to the most accurate cut-off value (>0.6387 ng/mL) identified by the Youden’s index. The results of this analysis demonstrated excellent discriminative ability, with an AUC of 0.9095 (95% CI = 0.8340–0.9851; *p* < 0.0001; std error = 0.03856; negative predictive power: 100%; positive predictive power: 86.36%), suggesting a high accuracy in differentiating between HSs and those with PD (Figure 2D). The overall model’s predictive performance was impressive, correctly classifying 90.32% of the cases.

## 3. Discussion

In this cross-sectional study, we compared the diagnostic value of oligomeric α-syn detection in saliva and the detection of skin p-S129 α-syn deposits in discriminating between 38 PD patients and 24 HSs. We confirmed that both techniques individually are capable of accurately distinguishing between PD patients and HSs. In addition, we found that there is modest degree of agreement between them. Finally, the combination of the two methods has an excellent ability in discriminating PD patients from HSs. We took all the precautions to avoid potential confounding factors. To ensure the accuracy and reliability of our PD patient cohort, we strictly recruited individuals who were diagnosed based on clinically established criteria [38] and with a disease duration exceeding 3 years. This approach minimized the risk of including misdiagnosed or atypical parkinsonian cases. Moreover, we meticulously adhered to previously established collecting protocols for both skin biopsies and salivary samples [9,11,12,25,27]. By following these standardized methods, we aimed at maintaining consistency in our data collection process, minimizing variability that could arise from non-standardized sampling procedures. Finally, to avoid analytical and data interpretation biases, standardized clinical scales were employed and administered by experienced movement disorder specialists, and evaluations of skin biopsies were performed by neurologists specialized in pain and small fibers neuropathies, following standardized methodologies [9,11].

### 3.1. Salivary Oligomeric α-syn in PD Patients

In our study, PD patients exhibited significantly higher mean levels of salivary oligomeric α-syn compared to HSs, corroborating findings coming from previous studies using similar methodological approaches on different biological fluids, including saliva [9,11,12,23,24], plasma [21] or CSF [39]. The detection of elevated salivary oligomeric α-syn levels in PD patients as assessed by ELISA is especially noteworthy, as it highlights a potential diagnostic biomarker for PD derived from an easily accessible, non-invasively sampled biological fluid with a straightforward detection method. New emerging seeding assay techniques, such as RT-QuIC, have also provided indirect confirmation of our findings, showing that saliva exhibits a greater α-syn seeding capacity in PD patients compared to HSs [10,35]. Furthermore, this finding aligns with our understanding of the pathophysiology of PD, where the presence and continuous production of α-syn oligomers are considered key mechanisms for the disease spread and progression [40,41]. In our study, we observed some overlap in salivary oligomeric α-syn between PD patients and HSs. This finding could be attributed to the fact that α-syn oligomers might aggregate also in the saliva of HSs since they are molecularly unstable in solution [42]. Moreover, the presence of soluble α-syn aggregates in biological fluids varies also in the different stages of PD [12,43]. The inclusion of patients with different stages of PD in our cohort might provide another explanation for this observation.

### 3.2. Skin Biopsy-Based Biomarkers in PD Patients

In our study, by using skin biopsy analysis, we found that PD patients had a higher frequency of p-S129 α-syn deposits in dermal nerve bundles and autonomic fibers compared to HSs, consistent with previous studies [25,26,27]. Noticeably, PD patients also had lower intraepidermal nerve fiber density compared to HSs, as previously reported [44], suggesting that PD is associated with peripheral small nerve fiber damage of uncertain clinical significance. Patients with skin p-S129 α-syn deposits showed lower intraepidermal nerve fiber density compared to those without α-syn aggregates, suggesting that p-S129 α-syn deposition in the skin may contribute to PD-related small fiber pathology, as previously reported [26]. As a novel finding, we report that patients with higher levels of oligomeric α-syn in saliva contextually report an increased deposition of p-S129 α-syn in the autonomic fibers of the skin, supporting the view that salivary α-syn may reflect the presence of α-syn aggregates in the autonomic fibers innervating salivary glands [45]. Our findings also revealed that skin p-S129 α-syn deposit detection by indirect immunofluorescence had an exceptional diagnostic ability to distinguish PD patients from HSs.

### 3.3. Diagnostic Accuracy of the Combination of Salivary and Skin-Based Biomarkers

In the present paper, we verified whether the diagnostic yield of salivary and cutaneous biomarkers in discriminating PD patients from HSs could be increased by combining the two methods’ results. This investigation was also motivated by the observation that some patients showed positivity for one biomarker but not for the other, and vice versa, indicating that α-syn oligomers in the saliva and α-syn aggregates in the skin may represent distinct aspects of α-syn pathology in PD and therefore suggesting that a combined approach might provide a higher diagnostic precision. The integration of the two tests, i.e., the presence of skin p-S129 α-syn deposits associated with the detection of higher salivary oligomeric α-syn levels, with respect to the best cut-off value (>0.6387 ng/mL), demonstrated a significant advancement in the non-invasive diagnosis of PD. Our age and gender-adjusted logistic regression model underscores the excellent discriminative capacity of these combined biomarkers, as evidenced by an AUC of 0.9095, with a remarkable precision in classification, accurately identifying 90.32% of the cases. In accordance, previous studies have shown that the employment of multiple α-syn-related biomarkers in a single biological tissue or fluid increases the diagnostic accuracy with respect to single methodologies [9,11,12,46]. Our findings suggest that the combination of different α-syn detection techniques for distinct biological tissues and fluids may be a valuable approach to enhance the diagnostic accuracy for α-syn related pathologies. Further studies are needed to identify in which class of patients one approach is more efficient than another, for diagnostic confirmation as well as for molecular follow up. In this regard, it is worth noting that saliva sampling is completely non-invasive, in comparison to the minimally invasive procedure used for skin biopsy. Besides the increased deposition of p-S129 α-syn in the skin of patients displaying higher levels of α-syn oligomers in the saliva (>1 ng/mL), we did not observe any correlation between salivary oligomeric α-syn levels and skin biopsy p-S129 α-syn-derived continuous measures, like the percentage of autonomic structures (sweat glands, piloerector muscles, blood vessels) or dermal nerve bundles showing positive staining. Similarly, there was no significant association between a pathological increase in salivary α-syn oligomers over the cut-off levels and skin biopsy positivity for p-S129 α-syn, and the agreement of the two techniques in discriminating PD patients from HSs was modest. This is likely attributable to the fact that salivary α-syn oligomers and p-S129 α -syn deposits in the skin likely represent different pathological signatures of α-syn misfolding and aggregation. Salivary α-syn oligomers are soluble α-syn aggregates detectable in biological fluids, with variable molecular conformation and biological properties [47]. Conversely, p-S129 α-syn deposits are formed by assembled phosphorylated α-syn filaments displaying an amyloid conformation and a high propensity to form insoluble intracellular aggregates [48,49,50,51]. Thus, α-syn oligomers and p-S129 α-syn aggregates might be differentially represented in distinct biological tissues and fluids, and their presence and concentration at different sites may vary according to disease duration and severity or patient clinical phenotype [43]. In this cohort, we failed to find correlations between the levels of salivary α-syn oligomers and the clinical scores of PD patients, corroborating observations coming from previous studies carried out by ELISA [9,11,12]. On the other hand, we observed that patients with cutaneous deposition of p-S129 α-syn exhibited higher scores on both the UPDRS III and NMSS compared to patients without p-S129 α-syn deposition. Recent studies have reported a direct association between p-S129 α-syn deposits in the skin and dysautonomia scores in PD patients [44,52], while we report, for the first time, a positive correlation with motor symptoms. These correlations possibly indicate that different degrees of p-S129 α-syn aggregation in the skin may contribute to the separation of different clinical phenotypes [53,54]. Interestingly, malignant clinical phenotypes, in addition to having greater motor severity, often manifest a higher non-motor burden, including autonomic disturbances such as orthostatic hypotension [53]. Our findings could thus suggest that the presence of p-S129 α-syn deposition in the skin may be associated with specific clinical features, potentially serving as a biomarker not only for diagnosing PD but also for assessing disease severity. However, further research is needed to confirm and gain a deeper understanding of the clinical significance of these correlations. Based on our findings showing a possible relation between skin deposits and disease severity and on previous studies suggesting that salivary oligomers may be more efficient as early biomarkers in the disease course [12], the modest diagnostic agreement between the two biomarkers could suggest that salivary α-syn oligomers and p-S129 α-syn deposits in the skin may achieve their maximum biomarker potential at different stages of PD and may be employed for different aspects of patient management.

### 3.4. Limitations

Our study, while providing useful insights, has some limitations that need to be considered. First, the relatively small sample size could limit the generalizability of our results. Future studies should aim to involve larger participant groups to enhance the reliability and applicability of these findings. Moreover, we recognize that the mean age of our control group did not exactly match that of the PD patients. However, due to the lack of correlation between age and our major outcome variables from salivary and skin analyses, this discrepancy is unlikely to affect the reliability of our findings. In addition, we adjusted the logistic regression models to consider age as a covariate. Another potential limitation is the cross-sectional nature of our study. While providing a valuable snapshot of biomarker levels and their diagnostic potential at a single point in time, this approach inherently limits our ability to draw conclusions about the progression of these biomarkers over the course of the disease. Finally, the varying stages of disease progression in our patient cohort present another level of complexity. PD is a condition that evolves over time, and the stage of disease could significantly influence biomarker levels. Future research might consider stratifying patients based on their disease stage (i.e., de-novo, early, moderate and advanced stages) to obtain more detailed and stage-specific insight.

## 4. Materials and Methods

### 4.1. Subjects

We consecutively enrolled 38 patients who received a diagnosis of idiopathic PD at the outpatient clinic of the Department of Human Neurosciences, Sapienza University of Rome. Diagnosis was established according to the International Parkinson and Movement Disorder Society clinical diagnostic criteria for PD [38]. A control group of 24 HSs, comprising both non-consanguineous caregivers of PD patients and healthy volunteers, was also enrolled. Control subjects underwent a thorough preliminary evaluation, which included a comprehensive medical history and a detailed neurological examination conducted by a neurologist, to rigorously exclude any existing neurological conditions. All subjects provided written informed consent for study participation. The protocol was approved by the ethical committee of the Policlinico Umberto I Hospital (n. 5433, prot n. 548/19) and performed in accordance with the Declaration of Helsinki.

### 4.2. Clinical Assessment

In all patients, a comprehensive medical history and neurological examination were conducted to identify any neurological conditions other than PD. Patients with co-existing neurological disorders were excluded from the study. Sociodemographic data, including sex, age, age at disease onset, disease duration and education level, were systematically collected. Information regarding current pharmacological treatments was also obtained, and the levodopa equivalent daily dose (LEDD) was calculated. Patients who had received infusion therapy or undergone surgical interventions for PD were not included in the study. Neurological assessment included the evaluation of disease staging using the Hoehn and Yahr scale (H&Y) [55]. The severity of motor symptoms was quantified utilizing the Italian version of the Movement Disorder Society-sponsored revision of the Unified Parkinson’s Disease Rating Scale (MDS-UPDRS) part III [56]. Motor complications, including off periods and dyskinesia, were assessed via the MDS-UPDRS Part IV [56]. The MDS-UPDRS Parts I and II were employed to evaluate the influence of motor and non-motor symptoms on daily living activities [56]. The severity of non-motor symptoms was evaluated by using the Non-Motor Symptoms Scale for PD (NMSS) [57]. This scale evaluates different domains related to cardiovascular symptoms, sleep disturbances, autonomic dysfunction, cognitive impairment, neuropsychiatric dysfunction and pain. Finally, the Montreal Cognitive Assessment (MoCA) was used to evaluate the presence of cognitive impairment and assess global cognitive functioning [58].

### 4.3. Salivary Sample Collection

Salivary samples were collected following established protocols from previous studies [9,11,12]. Detailed anamnestic information regarding oral health status was obtained for each participant before saliva collection. Subjects were instructed to abstain from eating, drinking and smoking for specific durations (2 h for food and drink, 4 h for smoking) and avoid alcohol for 12 h prior to saliva collection, with self-washing of the oral cavity done 1 h before. The collection process included an initial inspection of the oral cavity to exclude any pathologies, followed by the collection of saliva through drooling into a 50 mL vial, targeting at least 2 mL per subject. The saliva was then transferred to 10 mL Falcon tubes and immediately placed on dry ice, with the addition of a protease inhibitor (Sigma Aldrich, P2714) at a specified concentration. This was followed by centrifugation at 5000× *g* at 4 °C for 20 min to remove residual particles, and the resulting supernatant was aliquoted into 1 mL low-binding Eppendorf tubes for storage at −80 °C until ELISA analysis.

### 4.4. Competitive ELISA Analysis for Alpha-Synuclein Oligomers

The level of oligomeric α-syn was considered the main salivary analysis outcome variable. Prior to conducting the ELISA analysis, the total protein content of each saliva sample was determined using a BCA Protein Assay Kit (Thermofisher Scientific, Heysham, UK). This step was followed by normalizing the protein levels of saliva samples from PD patients and HSs using 0.001 M phosphate-buffered saline (PBS) to ensure uniform protein concentrations. To mitigate the potential impact of blood contamination on α-syn levels in saliva, we measured the hemoglobin content. Samples with hemoglobin concentrations exceeding 200 ng/mL were not included in the ELISA analysis. A Human A-SYN Competitive Oligomer ELISA Kit (MyBioSource, San Diego, CA, USA, MBS730762) was employed for quantifying α-syn oligomers in saliva. In this process, saliva was diluted at a 1:1 ratio with 0.001 M PBS, and the conjugate antibody was incubated for 3 h at 37 °C, with the remaining procedures being in line with the manufacturer’s guidelines. A spectrometric assessment at 450 nm wavelength in an appropriate microplate reader (LT 4000, Labtech, Heathfield, Tonbridge, UK) was performed to determine the concentration of α-syn oligomers in saliva. Standard curves were generated by correlating the absorbance or optical density measurements of the standards with their respective concentrations.

### 4.5. Skin Biopsy Sample Collection

All patients and HSs underwent skin biopsy at a cervical site, C7 level, 2 cm laterally on the right-side respect to the dorsal spine line, using a 3 mm disposable circular punch after local lidocaine anesthesia under sterile conditions, with no suture requirement [26,59]. Biopsies were fixed for 24 h at 4 °C in Zamboni’s fixative and then cryoprotected overnight. Cutting was performed at −23 °C with a cryostat (MEV, SLEE medical, Nieder-Olm, Germany) to obtain both 50 and 10 micron thick sections.

### 4.6. Intraepidermal Nerve Fiber Density Assessment

Intraepidermal innervation was assessed with the pan-neuronal marker PGP9.5 by indirect immunofluorescence. From each sample, three non-consecutive, 50 µm thick sections were selected for immunostaining and blocked with 5% normal donkey serum for 1 h. Sections were incubated overnight with a rabbit anti-human PGP9.5 polyclonal antibody (Abcam, Waltham, MA, USA, ab108986, 1:500 diluted) and a mouse anti-human collagen IV monoclonal antibody (Millipore, Burlington, MA, USA, MAB1910, 1:1600). The following day, sections were incubated overnight with anti-rabbit-Cy3 (Jakson, West Grove, PA, USA, 711-165-152, 1:800) and anti-mouse-488 (Jakson, 715-545-150, 1:400) secondary antibodies. Intraepidermal nerve fibers were counted under a fluorescence microscope (Leyca NB) with appropriate wavelength filters. The epidermal linear length of each skin section was measured through Image J to obtain the linear density. IENFD was calculated according to the guidelines of the European Federation of Neurological Societies and Peripheral Nerve Society [43] and expressed as number of fibers/mm.

### 4.7. P-S129 Alpha-Synuclein Deposits Evaluation and Quantification

To achieve an accurate and thorough histological analysis, in our study, we analyzed four 10 µm thick skin sections plus four 50 µm thick skin sections for each patient, derived from random sampling of the skin biopsy. In fact, though most studies used 10 µm thick sections, it has been suggested that a higher thickness, like 50 µm, increases the probability of detecting p-S129 α-syn aggregates [60]. Skin sections were blocked with 5% normal donkey serum for 1 h and immuno-stained overnight with a monoclonal rabbit primary antibody for p-S129 α-syn (Abcam, ab51253, 1:500) and a monoclonal mouse antibody for PGP9.5 (Abcam,1:750). Sections were then washed and incubated for 2 h with anti-rabbit-Cy3 (Jakson, 711-165-152, 1:200) and anti-mouse-488 (Jakson, 715-545-150, 1:200) secondary antibodies. Sections were analyzed under the fluorescent microscope. The analysis was carried out independently by two blinded operators with expertise in immunofluorescence analysis (EG, GV). These two operators were in full agreement on the positivity or negativity of the phosphorylated α-synuclein staining without any uncertain classification. Each sample was considered positive for p-S129 α-syn detection if at least one out of eight sections showed deposits in at least one autonomic structure (i.e., sweat gland, piloerector muscle, blood vessel) or nerve bundle. Deposits were considered positive if colocalizing with PGP9.5 immunostaining. The positivity of p-S129 α-syn detection was considered the main diagnostic variable for skin biopsy analysis. p-S129 α-syn staining was rated as the percentage of autonomic structures (sweat glands, piloerector muscles, blood vessels) and dermal nerve bundles showing positive staining at high magnification (40×) in each section [61]. The value for each individual represented a mean of the eight skin sections.

### 4.8. Statistical Analysis

Statistical analysis was performed using Prism GraphPad version 10.1.1 (Boston, MA, USA). Continuous data were reported as means ± standard deviations. The Shapiro–Wilk test was used to determine whether all variables fit a normal distribution. Depending on the data distribution, both the parametric Student *t*-test and non-parametric Mann–Whitney U test were used to compare salivary and skin biomolecular parameters between the groups (healthy controls vs. PD patients). The main clinical variables were compared between patients with and without p-S129 α-syn skin deposition. To explore correlations between variables, multiple Spearman’s rank correlation coefficients were applied. We examined correlations among continuous α-syn-related diagnostic variables in skin and saliva and between these parameters and the clinical scores of PD patients. Age and gender-adjusted logistic regression analyses were used to determine if salivary or skin biomarkers could predict disease status. Additionally, receiver operating characteristic (ROC) curve analysis and Youden’s index were applied to further assess the diagnostic performance of these biomarkers in discriminating between PD patients and HSs. The agreement between the two procedures for α-syn detection was evaluated by means of Cohen’s kappa index. A value of *p* < 0.05 indicated statistical significance. FDR correction was used to correct for multiple comparisons where appropriate.

## 5. Conclusions

In conclusion, beyond confirming the diagnostic yield of salivary and skin-based biomarkers, our study findings suggest that a combined approach, utilizing multiple accessible tissue-based biomarkers, is highly performative in discriminating PD patients from HSs and may significantly improve PD diagnosis. Moreover, our study suggests that different sources of biomarkers may display a maximized diagnostic potential at distinct disease stages in different classes of patients. This research significantly contributes to the expanding body of knowledge, advancing the development of accessible diagnostic methods for PD. Importantly, it lays the foundation for the development of future studies with extensive case cohorts and longitudinal designs, enabling the validation of the practical applicability of these biomarkers within clinical settings.

## Figures and Tables

**Table 1:** Clinical and demographic characteristics of PD patients and healthy subjects.

Variable	PD Patients (*n* = 38)	Healthy Subjects (*n* = 24)
Age (mean ± SD)	72.1 ± 8.4	59.5 ± 16.9
Sex	11 F, 27 M	12 F, 12 M
Age at onset (mean ± SD)	66.9 ± 8.7	-
Disease duration (mean ± SD)	5.2 years ± 4.4	-
MDS-UPDRS Part I (mean ± SD)	8.7 ± 6.48	-
MDS-UPDRS Part II (mean ± SD)	7.7 ± 7.99	-
MDS-UPDRS Part III (mean ± SD)	22.6 ± 13.7	-
MDS-UPDRS Part IV (mean ± SD)	1.66 ± 3.7	-
NMSS total score (mean ± SD)	45.16 ± 35.87	-
MoCA (mean ± SD)	24.59 ± 3.83	-
H&Y (mean ± SD)	1.78 ± 0.66	-
LEDDs (mean ± SD)	427.3 ± 247.4	-
Dyskinesias	7/38 (18.4%)	-
Motor fluctuations	7/38 (18.4%)	-

Continuous variables are expressed as mean and standard deviations (SD). Categorical variables are expressed as number of subjects (n) and percentages (%). Abbreviations: MDS-UPDRS: Movement Disorder Society-sponsored revision of the Unified Parkinson’s Disease Rating Scale; NMSS: non-motor symptoms scale for PD; MoCA: Montreal Cognitive Assessment; H&Y: Hoehn and Yahr scale; LEDDs: levodopa equivalent daily dose.

**Table 2:** Skin biopsy and salivary variables in patients with Parkinson’s disease and healthy subjects.

	PD Patients (38)	Healthy Subjects (24)	*p*-Value
IENFD, n fibers/mm, mean ± SD	20.2 ± 7.8	28.7 ± 10.6	*p* = 0.0008
Autonomic structures p-S129-syn positivity, mean % ± SD	4 ± 10	0 ± 0	*p* < 0.0001
Dermal nerve bundles p-S129-syn positivity, % ±SD	9 ± 14	0.5 ± 2	*p* < 0.0001
p-S129-syn positivity, n (%)	26 (68%)	2 (8%)	*p* < 0.0001
p-S129-syn positivity on 50 µ thick sections, n (%)	22 (58%)	2 (8%)	*p* < 0.0001
p-S129-syn positivity on 10 µ thick sections, n (%)	17 (45%)	1 (4%)	*p* < 0.0001
Salivary oligomeric α-syn, mean ± SD (ng/mL)	1.17 ± 0.90	0.7 ± 0.6	*p* = 0.003

Continuous variables are expressed as mean and standard deviations (SD). Categorical variables are expressed as number of subjects (n) and percentages (%). Continuous variables were compared by Mann–Whitney U test. Categorical variables were compared by Fisher’s exact test. Abbreviations: IENFD: intraepidermal nerve fiber density; Sweat gland p-S129 α-syn positivity: percentage of sweat glands showing p-S129 α-syn deposits; Piloerector muscle p-S129 α-syn positivity: percentage of piloerector muscles showing p-S129 α-syn deposits; Autonomic structures p-S129 α-syn positivity: percentage of sweat glands and/or piloerector muscles showing p-S129 α-syn deposits in eight skin sections (50 and 10 µ thick sections); Dermal nerve bundles p-S129 α-syn positivity: percentage of dermal nerve bundles showing p-S129 α-syn deposits in eight skin sections (50 and 10 µ thick sections); p-S129 α-syn positivity: percentage of subjects showing p-S129 α-syn positivity on at least one autonomic structure or dermal nerve bundle in eight skin sections (50 and 10 µ thick sections); p-S129 α-syn positivity for 50 and 10 µ thick sections are then reported separately.

## Data Availability

The data supporting the findings of this study are available upon request from the corresponding author. The data are not publicly available due to privacy or ethical restrictions.

## Supplementary Materials

The following supporting information can be downloaded at: https://www.mdpi.com/article/10.3390/ijms25094823/s1.
