# Supplementary material for: Evaluating the Diagnostic Potential of Combined Salivary and Skin Biomarkers in Parkinson’s Disease
Source: Int J Mol Sci. 2024 Apr 28;25(9):4823. doi: 10.3390/ijms25094823 (PMC11084721; doi:10.3390/ijms25094823)
Supplement: Supplementary file 1 [file ijms-25-04823-s001.zip › ijms-2953512-supplementary.pdf]

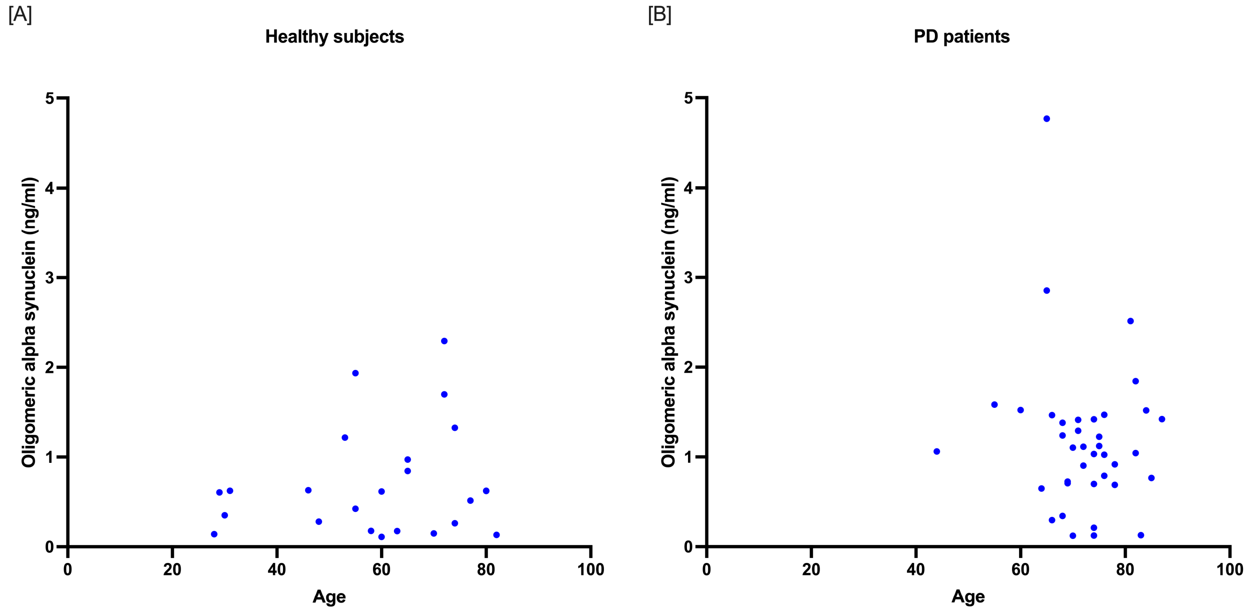

Figure S1. Relationship between salivary oligomeric alpha synuclein (expressed in ng/ml) and age in healthy subjects (A) and PD patients (B)

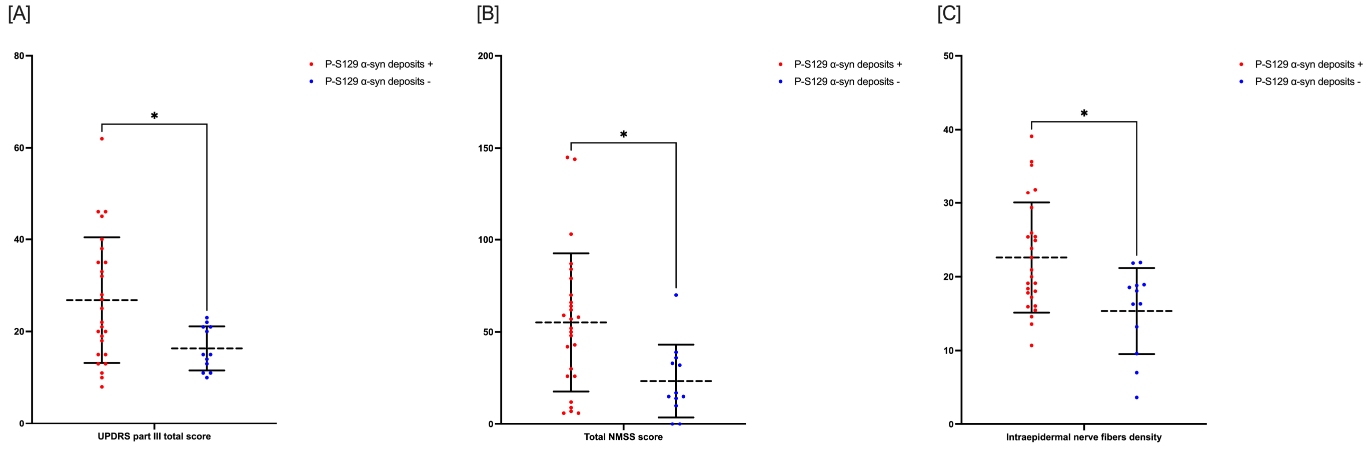

Figure S2. Comparison between PD patients who exhibit deposits of p-S129 alpha synuclein in the skin and those without such deposits in terms of total score on the MDS-UPDRS (A), NMSS (B) and intraepidemal nerve fibre density (C). Mean values (dottel lines) and standard deviations (error bars) are shown in the figure. The asterisk (*) indicates statistical significance.

Abbreviations: UPDRS: Unified Parkinson's disease rating scale; NMSS: Non-motor symptoms assessment scale

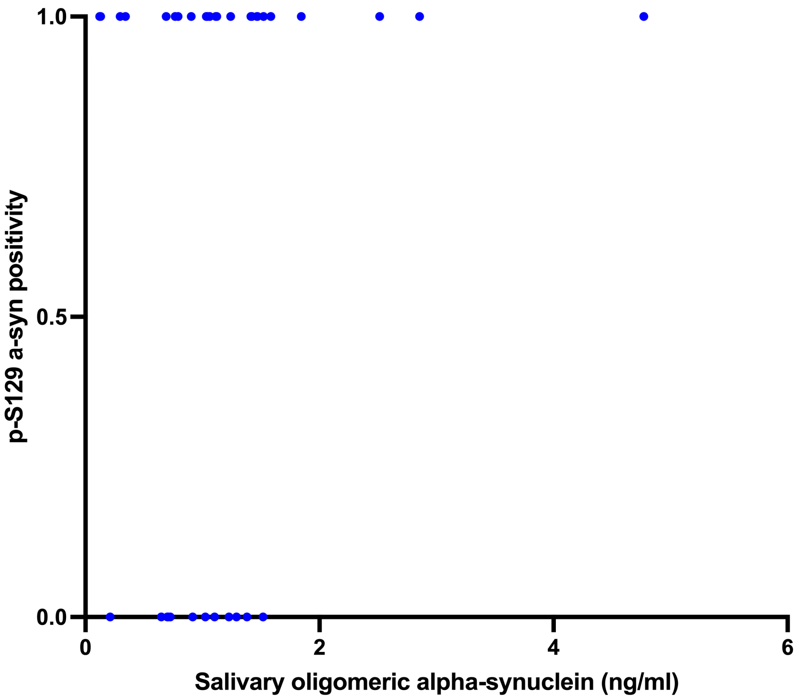

Figure S3. Scatter plot illustrating the relationship between salivary oligomeric alpha synuclein (expressed in ng/ml) and skin positivity of p-S129 alpha synuclein

Model	Variable	Estimate (odd ratios)	95% CI (profile likelihood)	p value	R squared	AUC	Negative predictive power (%)	Positive predictive power (%)
1	Gender (M:1; F:0)	2.169	0,6408 to 7,505	0,0003	0,2821	0,7752	78,95	79,07
	Age	1,080	1,026 to 1,153					
	Oligomeric alpha syn saliva (ng/ml)	2,093	0,8700 to 6,397					
2	Gender (M:1; F:0)	1,685	0,4131 to 6,882	<0,0001	0,4302	0,8750	68,18	77,50
	Age	1,082	1,022 to 1,169					
	Skin p-S129 alpha synuclein positivity	17,29	3,748 to 133,7					
3	Gender (M:1; F:0)	1,993	0,4848 to 8,315	<0,0001	0,4434	0,8487	78,26	84,62
	Age	1,059	1,001 to 1,136					
	Youden's index-based positivity of salivary alpha synuclein oligomers	12,68	3,318 to 56,23					
4	Gender (M:1; F:0)	1,264	0,2175 to 6,812	<0,0001	0,6139	0,9095	100,0	86,36
	Age	1,062	0,9660 to 1,197					
	Skin p-S129 alpha synuclein positivity	34,08	4,807 to 755,7					
	Youden's index-based positivity of salivary alpha synuclein oligomers	28,90	4,415 to 578,6					

Table S1. Age and gender – adjusted logistic regression models
